# Supplementary material for: Retrospective study of more than 5 million emergency admissions to hospitals in England: Epidemiology and outcomes for people with dementia
Source: PLoS One. 2023 Mar 8;18(3):e0281158. doi: 10.1371/journal.pone.0281158 (PMC9994676; doi:10.1371/journal.pone.0281158)
Supplement: S1 File — (DOCX) [file pone.0281158.s002.docx]

**S1 File. Supplementary materials**

| **Box A1. ICD10 codes used to identify a diagnosis of dementia** | |  |
| --- | --- | --- |
| **A81.0** | **Creutzfeldt-Jakob disease** |  |
|  | Subacute spongiform encephalopathy |  |
| **F00*** | **Dementia in Alzheimer's disease** |  |
| **F00.0*** | **Dementia in Alzheimer's disease with early onset** |  |
|  | Alzheimer's disease, type 2  Presenile dementia, Alzheimer's type  Primary degenerative dementia of the Alzheimer's type, presenile onset |  |
| **F00.1*** | **Dementia in Alzheimer's disease with late onset** |  |
|  | Alzheimer's disease, type 1  Primary degenerative dementia of the Alzheimer's type, senile onset  Senile dementia, Alzheimer's type |  |
| **F00.2*** | **Dementia in Alzheimer's disease, atypical or mixed type** |  |
|  | Atypical dementia, Alzheimer's type |  |
| **F00.9*** | **Dementia in Alzheimer's disease, unspecified** |  |
| **F01** | **Vascular dementia** |  |
| **F01.0** | **Vascular dementia of acute onset** |  |
| **F01.1** | **Multi-infarct dementia** |  |
| **F01.2** | **Subcortical vascular dementia** |  |
| **F01.3** | **Mixed cortical and subcortical vascular dementia** |  |
| **F01.8** | **Other vascular dementia** |  |
| **F01.9** | **Vascular dementia, unspecified** |  |
| **F02*** | **Dementia in other diseases classified elsewhere** |  |
| **F02.0*** | **Dementia in Pick's disease** |  |
| **F02.1*** | **Dementia in Creutzfeldt-Jakob disease** |  |
| **F02.2*** | **Dementia in Huntington's disease** |  |
| **F02.3*** | **Dementia in Parkinson's disease** |  |
|  | Dementia in:  · paralysis agitans  · parkinsonism | |
| **F02.4*** | **Dementia in human immunodeficiency virus [HIV] disease** | |
| **F02.8*** | **Dementia in other specified diseases classified elsewhere** | |
|  | Dementia in:  · cerebral lipidosis  · epilepsy  · hepatolenticular degeneration  · hypercalcaemia  · hypothyroidism, acquired  · intoxications  · multiple sclerosis  · neurosyphilis  · niacin deficiency [pellagra]  · polyarteritis nodosa  · systemic lupus erythematosus  · trypanosomiasis  · vitamin B _12_ deficiency | |
| **F03** | **Unspecified dementia** | |
|  | Presenile:  · dementia NOS  · psychosis NOS  Primary degenerative dementia NOS Senile:  · dementia:  · NOS  · depressed or paranoid type  · psychosis NOS | |
| **F04** | **Organic amnesic syndrome, not induced by alcohol and other psychoactive substances** | |
|  | Korsakov's psychosis or syndrome, nonalcoholic | |
| **F05.1** | **Delirium superimposed on dementia** | |
| **G30.0** | **Alzheimer's disease with early onset** | |
| **G30.1** | **Alzheimer's disease with late onset** | |
| **G30.8** | **Other Alzheimer's disease** | |
| **G30.9** | **Alzheimer's disease, unspecified** | |
| **G31.0** | **Circumscribed brain atrophy** | |
|  | Pick's disease  Progressive isolated aphasia | |
| **G31.1** | **Senile degeneration of brain, not elsewhere classified** | |
| **G31.8** | **Other specified degenerative diseases of nervous system** | |
|  | Grey-matter degeneration [Alpers]  Lewy body(ies)(dementia)(disease)  Subacute necrotizing encephalopathy [Leigh] | |
| **I67.3** | **Progressive vascular leukoencephalopathy** | |
|  | Binswanger's disease | |
| Note: On the advice of the study panel of clinical experts we excluded a few codes that had been included in the definition used by the National Audit of Dementia. These were codes F07.2, F10.6, F11.6, F13.6, F14.6, F15.6, F16.6, F17.6, F18.6, F19.6, which are not dementia per se, but neurological conditions related to other factors such as concussion, trauma, alcohol or drug use. | | |

| **Box A2. Details of the patient outcomes and explanatory factors used in the study** |
| --- |
| **Patient outcomes** |
| Length of hospital stay (LoS). Defined as the number of consecutive days between admission and subsequent discharge, including all consultant episodes during that period.  Long stay. A spell of 15 days or more. Applies to approximately 20% of all spells.  Emergency re-admission (ERA). An admission for an emergency within 30 days of a previous discharge.  Death. Defined as death while in hospital or within 30 days of discharge. We analysed total deaths and also sub-categories of deaths in hospital and deaths after discharge. Death was based on a flag in the dataset derived from Office for National Statistics mortality data.  ERA or death. Spells that end with a patient’s death cannot result in an ERA. Thus an increase/decrease in deaths may engender an apparently reduced/increased ERA rate. We therefore also analysed death or ERA as a combined outcome. |
| **Explanatory factors** |
| Demographic characteristics: age on admission (in 5-year age-bands); ethnicity (in 10 categories); residential area Index of Multiple Deprivation quintile (IMD 2010 for FY 2010/11; IMD 2015 for FYs 2012/13 and 2016/17); residential area low income quintile (% of the Lower Super Output Area (LSOA) population in receipt of pension credit or income support, expressed as a national quintile).  Spell characteristics: month of admission (in seasonal quarters); admitted on a weekend (yes or no); source of admission (usual residence, other hospital, residential care/care home, other/unknown).  Pre-existing health: The 30 Elixhauser comorbidities, based on the International Classification of Diseases version 10 (ICD-10) codes associated with each secondary diagnosis. A comorbidity was coded as present if an associated ICD-10 code was recorded for the current admission or any other admission over the present or previous financial year. We excluded codes for dementia in the construction of the comorbidity “other (non-dementia) neurodegenerative disorders”; numbers with AIDS/HIV were very small and were collapsed with lymphoma). Number of emergency admissions in the previous financial year (coded from 0 to 6, and 7 plus).  Admitting condition: the primary reason for admission and type of treatment given in the spell, recorded in the HES as the core Heath Resource Group (HRG) subchapter for the first episode in a spell. Subchapters applying to fewer than around 200 PwD were merged with closely related subchapters, or where a related subchapter did not exist combined into a catch-all “other” admissions category.  Number of consultant episodes: the number of different specialist consultants providing an episode of care to the patient during the spell, coded as 1, 2, 3, or 4 plus.  Discharge destination: The place to where the patient was discharged, coded as usual residence, other hospital, residential care/care home, died in hospital, other/unknown. Discharge destination was excluded from models where the outcome was death or death/ERA combined. |

| **Table A1. Descriptive statistics for the FY 2010/11 hospital spell sample** | | | | | | | | |
| --- | --- | --- | --- | --- | --- | --- | --- | --- |
|  | **Males** | | | | **Females** | | | |
|  | **PwD** |  | **PwoD** |  | **PwD** |  | **PwoD** |  |
|  | **n** | **%** | **n** | **%** | **n** | **%** | **n** | **%** |
| Total number of spells | 87,924 | 11.8 | 659,615 | 88.2 | 153,131 | 16.8 | 756,483 | 83.2 |
| **Patient demographics** |  |  |  |  |  |  |  |  |
| Age on admission |  |  |  |  |  |  |  |  |
| 65-69 | 3,714 | 4.2 | 125,418 | 19.0 | 3,044 | 2.0 | 105,677 | 14.0 |
| 70-74 | 8,107 | 9.2 | 136,464 | 20.7 | 7,547 | 4.9 | 123,301 | 16.3 |
| 75-79 | 15,705 | 17.9 | 139,561 | 21.2 | 18,540 | 12.1 | 142,603 | 18.9 |
| 80-84 | 23,358 | 26.6 | 126,645 | 19.2 | 34,992 | 22.9 | 154,002 | 20.4 |
| 85-89 | 23,884 | 27.2 | 89,273 | 13.5 | 47,914 | 31.3 | 135,673 | 17.9 |
| 90plus | 13,156 | 15.0 | 42,254 | 6.4 | 41,094 | 26.8 | 95,227 | 12.6 |
| Mean (SD) | 82.6 | 6.9 | 77.3 | 7.7 | 85.3 | 6.7 | 79.6 | 8.3 |
| Ethnicity |  |  |  |  |  |  |  |  |
| White | 79,992 | 91.0 | 593,724 | 90.0 | 140,985 | 92.1 | 686,792 | 90.8 |
| Black African | 188 | 0.2 | 1,656 | 0.3 | 222 | 0.1 | 1,666 | 0.2 |
| Black Caribbean | 1,180 | 1.3 | 5,916 | 0.9 | 1,108 | 0.7 | 5,400 | 0.7 |
| Indian | 849 | 1.0 | 8,753 | 1.3 | 965 | 0.6 | 8,731 | 1.2 |
| Pakistani | 510 | 0.6 | 5,768 | 0.9 | 381 | 0.2 | 4,917 | 0.6 |
| Bangladeshi | 166 | 0.2 | 2,030 | 0.3 | 89 | 0.1 | 1,175 | 0.2 |
| Black other/mixed | 259 | 0.3 | 1,668 | 0.3 | 232 | 0.2 | 1,634 | 0.2 |
| Asian other/mixed | 354 | 0.4 | 3,265 | 0.5 | 356 | 0.2 | 2,959 | 0.4 |
| Other ethnic group/mixed | 855 | 1.0 | 6,717 | 1.0 | 1,354 | 0.9 | 6,847 | 0.9 |
| Unknown | 3,571 | 4.1 | 30,118 | 4.6 | 7,439 | 4.9 | 36,362 | 4.8 |
| Residential IMD quintile (2015) |  |  |  |  |  |  |  |  |
| 1 | 19,391 | 22.1 | 132,458 | 20.1 | 32,743 | 21.4 | 154,782 | 20.5 |
| 2 | 18,450 | 21.0 | 131,527 | 19.9 | 33,109 | 21.6 | 155,525 | 20.6 |
| 3 | 18,110 | 20.6 | 137,408 | 20.8 | 32,006 | 20.9 | 157,885 | 20.9 |
| 4 | 16,890 | 19.2 | 133,889 | 20.3 | 29,540 | 19.3 | 149,852 | 19.8 |
| 5 | 14,345 | 16.3 | 118,127 | 17.9 | 24,541 | 16.0 | 131,929 | 17.4 |
| Missing | 738 | 0.8 | 6,206 | 0.9 | 1,192 | 0.8 | 6,510 | 0.9 |
| Residential area low income quintile |  |  |  |  |  |  |  |  |
| 1 | 13,143 | 14.9 | 124,037 | 18.8 | 21,477 | 14.0 | 130,411 | 17.2 |
| 2 | 15,366 | 17.5 | 123,615 | 18.7 | 25,646 | 16.7 | 136,588 | 18.1 |
| 3 | 16,827 | 19.1 | 124,712 | 18.9 | 30,589 | 20.0 | 145,816 | 19.3 |
| 4 | 18,865 | 21.5 | 127,454 | 19.3 | 34,402 | 22.5 | 154,212 | 20.4 |
| 5 | 20,945 | 23.8 | 137,710 | 20.9 | 36,412 | 23.8 | 165,281 | 21.8 |
| Missing | 2,778 | 3.2 | 22,087 | 3.3 | 4,605 | 3.0 | 24,175 | 3.2 |
| **Spell characteristics** |  |  |  |  |  |  |  |  |
| Month of admission |  |  |  |  |  |  |  |  |
| March-May | 20,440 | 23.2 | 157,209 | 23.8 | 35,814 | 23.4 | 177,415 | 23.5 |
| June-August | 22,435 | 25.5 | 162,256 | 24.6 | 38,450 | 25.1 | 186,969 | 24.7 |
| September-November | 23,115 | 26.3 | 167,477 | 25.4 | 40,340 | 26.3 | 193,332 | 25.6 |
| December-February | 21,934 | 24.9 | 172,673 | 26.2 | 38,527 | 25.2 | 198,767 | 26.3 |
| Weekend admission (Sat or Sun) |  |  |  |  |  |  |  |  |
| Yes | 23,315 | 26.5 | 154,105 | 23.4 | 41,880 | 27.3 | 180,104 | 23.8 |
| No | 64,609 | 73.5 | 505,510 | 76.6 | 111,251 | 72.7 | 576,379 | 76.2 |
| Source of admission |  |  |  |  |  |  |  |  |
| Usual residence | 81,317 | 92.5 | 625,506 | 94.8 | 141,144 | 92.2 | 716,336 | 94.7 |
| Other hospital | 4,500 | 5.1 | 30,067 | 4.6 | 7,922 | 5.2 | 34,722 | 4.6 |
| Residential care/care home | 1,808 | 2.1 | 1,567 | 0.2 | 3,588 | 2.3 | 2,524 | 0.3 |
| Other/unknown | 299 | 0.3 | 2,475 | 0.4 | 477 | 0.3 | 2,901 | 0.4 |
| **Pre-existing health** |  |  |  |  |  |  |  |  |
| Elixhauser comorbidities |  |  |  |  |  |  |  |  |
| Congestive heart failure | 14,836 | 16.9 | 122,005 | 18.5 | 23,727 | 15.5 | 125,362 | 16.6 |
| Cardiac arrhythmias | 32,852 | 37.4 | 229,020 | 34.7 | 48,749 | 31.8 | 230,934 | 30.5 |
| Valvular disease | 6,047 | 6.9 | 62,076 | 9.4 | 10,757 | 7.0 | 70,140 | 9.3 |
| Pulmonary circulation disorders | 1,807 | 2.1 | 24,912 | 3.8 | 3,387 | 2.2 | 30,010 | 4.0 |
| Peripheral vascular disorders | 7,381 | 8.4 | 69,734 | 10.6 | 6,816 | 4.5 | 42,696 | 5.6 |
| Hypertension | 45,175 | 51.4 | 375,828 | 57.0 | 81,562 | 53.3 | 451,752 | 59.7 |
| Paralysis | 4,084 | 4.6 | 21,815 | 3.3 | 5,214 | 3.4 | 19,674 | 2.6 |
| Other (non-dementia) neurodegenerative disorders | 21,682 | 24.7 | 57,668 | 8.7 | 24,743 | 16.2 | 56,891 | 7.5 |
| Chronic pulmonary disease | 19,455 | 22.1 | 186,717 | 28.3 | 26,242 | 17.1 | 197,361 | 26.1 |
| Diabetes, uncomplicated | 20,275 | 23.1 | 155,160 | 23.5 | 27,494 | 18.0 | 144,009 | 19.0 |
| Diabetes, complicated | 2,417 | 2.7 | 20,816 | 3.2 | 2,146 | 1.4 | 14,743 | 1.9 |
| Hypothyroidism | 5,250 | 6.0 | 31,882 | 4.8 | 22,336 | 14.6 | 105,740 | 14.0 |
| Renal failure | 16,692 | 19.0 | 104,262 | 15.8 | 20,741 | 13.5 | 89,646 | 11.9 |
| Liver disease | 1,774 | 2.0 | 19,261 | 2.9 | 1,671 | 1.1 | 15,863 | 2.1 |
| Peptic ulcer disease excluding bleeding | 1,402 | 1.6 | 12,848 | 1.9 | 1,408 | 0.9 | 10,914 | 1.4 |
| Lymphoma | 626 | 0.7 | 10,831 | 1.6 | 674 | 0.4 | 8,849 | 1.2 |
| Metastatic cancer | 2,191 | 2.5 | 45,340 | 6.9 | 1,852 | 1.2 | 34,950 | 4.6 |
| Solid tumour without metastasis | 10,131 | 11.5 | 108,602 | 16.5 | 5,946 | 3.9 | 63,017 | 8.3 |
| Rheumatoid arthritis/collagen vascular diseases | 2,216 | 2.5 | 25,094 | 3.8 | 7,101 | 4.6 | 55,478 | 7.3 |
| Coagulopathy | 969 | 1.1 | 9,236 | 1.4 | 1,084 | 0.7 | 7,634 | 1.0 |
| Obesity | 724 | 0.8 | 14,977 | 2.3 | 1,222 | 0.8 | 18,916 | 2.5 |
| Weight loss | 2,582 | 2.9 | 21,115 | 3.2 | 3,439 | 2.2 | 21,153 | 2.8 |
| Fluid and electrolyte disorders | 21,388 | 24.3 | 87,852 | 13.3 | 36,618 | 23.9 | 112,182 | 14.8 |
| Blood loss anemia | 202 | 0.2 | 1,798 | 0.3 | 262 | 0.2 | 1,683 | 0.2 |
| Deficiency anemia | 6,402 | 7.3 | 38,445 | 5.8 | 12,156 | 7.9 | 52,361 | 6.9 |
| Alcohol abuse | 3,814 | 4.3 | 36,166 | 5.5 | 2,219 | 1.4 | 13,648 | 1.8 |
| Drug abuse | 81 | 0.1 | 628 | 0.1 | 99 | 0.1 | 473 | 0.1 |
| Psychoses | 1,503 | 1.7 | 3,977 | 0.6 | 2,731 | 1.8 | 5,294 | 0.7 |
| Depression | 8,222 | 9.4 | 29,336 | 4.4 | 17,808 | 11.6 | 52,266 | 6.9 |
| Number of Elixhauser comorbidities |  |  |  |  |  |  |  |  |
| Mean (SD) | 3.0 | 2.0 | 2.9 | 2.0 | 2.6 | 1.9 | 2.7 | 1.9 |
| No. of emergency admissions in previous FY |  |  |  |  |  |  |  |  |
| None | 45,262 | 51.5 | 452,769 | 68.6 | 83,890 | 54.8 | 523,496 | 69.2 |
| 1 | 20,206 | 23.0 | 113,555 | 17.2 | 35,600 | 23.2 | 132,670 | 17.5 |
| 2 | 10,353 | 11.8 | 45,378 | 6.9 | 17,147 | 11.2 | 52,478 | 6.9 |
| 3 | 5,342 | 6.1 | 21,101 | 3.2 | 7,883 | 5.1 | 22,732 | 3.0 |
| 4 | 2,586 | 2.9 | 10,546 | 1.6 | 4,093 | 2.7 | 10,971 | 1.5 |
| 5 | 1,600 | 1.8 | 5,799 | 0.9 | 1,935 | 1.3 | 5,569 | 0.7 |
| 6 | 1,030 | 1.2 | 3,246 | 0.5 | 1,122 | 0.7 | 3,201 | 0.4 |
| 7+ | 1,545 | 1.8 | 7,221 | 1.1 | 1,461 | 1.0 | 5,366 | 0.7 |
| **Admitting condition type and severity** |  |  |  |  |  |  |  |  |
| Spell Core HRG subchapter |  |  |  |  |  |  |  |  |
| AA+AB Nervous System Procedures and Disorders/Pain management | 7,406 | 8.4 | 48,293 | 7.3 | 12,212 | 8.0 | 55,659 | 7.4 |
| BZ Eyes and Periorbita Procedures and Disorders | 274 | 0.3 | 2,877 | 0.4 | 673 | 0.4 | 3,535 | 0.5 |
| CZ Mouth Head Neck and Ears Procedures and Disorders | 2,619 | 3.0 | 17,472 | 2.6 | 5,715 | 3.7 | 20,647 | 2.7 |
| DZ Respiratory System Procedures and Disorders | 15,724 | 17.9 | 110,548 | 16.8 | 20,530 | 13.4 | 111,401 | 14.7 |
| EA Cardiac Procedures | 629 | 0.7 | 18,841 | 2.9 | 558 | 0.4 | 12,544 | 1.7 |
| EB Cardiac Disorders | 10,303 | 11.7 | 117,036 | 17.7 | 16,995 | 11.1 | 126,786 | 16.8 |
| FZ Digestive System Procedures and Disorders | 7,407 | 8.4 | 76,955 | 11.7 | 13,053 | 8.5 | 94,956 | 12.6 |
| GA+GB Hepatobiliary and Pancreatic System Open, Laparoscopic, Endoscopic Procedures | 286 | 0.3 | 6,159 | 0.9 | 416 | 0.3 | 6,573 | 0.9 |
| GC Hepatobiliary and Pancreatic System Disorders | 490 | 0.6 | 10,027 | 1.5 | 841 | 0.5 | 11,052 | 1.5 |
| HA Orthopaedic Trauma Procedures | 3,434 | 3.9 | 16,918 | 2.6 | 13,729 | 9.0 | 50,231 | 6.6 |
| HB+HR Orthopaedic Non-Trauma + Reconstruction Procedures | 544 | 0.6 | 6,728 | 1.0 | 1,055 | 0.7 | 7,774 | 1.0 |
| HC Spinal Procedures and Disorders | 788 | 0.9 | 8,748 | 1.3 | 1,573 | 1.0 | 12,610 | 1.7 |
| HD Musculoskeletal and Rheumatological Disorders | 3,165 | 3.6 | 24,554 | 3.7 | 9,019 | 5.9 | 42,897 | 5.7 |
| JA+JB+JC Breast/Burn/Skin Procedures and Disorders | 794 | 0.9 | 6,303 | 1.0 | 1,678 | 1.1 | 8,318 | 1.1 |
| JD Skin Disorders | 1,232 | 1.4 | 12,452 | 1.9 | 2,927 | 1.9 | 15,670 | 2.1 |
| KA+KC Endocrine System/Metabolic Disorders | 1,200 | 1.4 | 6,648 | 1.0 | 2,915 | 1.9 | 9,454 | 1.2 |
| KB Diabetic Medicine | 966 | 1.1 | 5,363 | 0.8 | 1,546 | 1.0 | 5,165 | 0.7 |
| LA Renal Procedures and Disorders | 9,139 | 10.4 | 29,639 | 4.5 | 16,609 | 10.8 | 38,586 | 5.1 |
| LB Urological and Male Reproductive System Procedures and Disorders | 4,911 | 5.6 | 38,705 | 5.9 | 927 | 0.6 | 7,144 | 0.9 |
| MA+MB Female Reproductive System Procedures/Disorders | NA | NA | NA | NA | 446 | 0.3 | 3,631 | 0.5 |
| QZ Vascular Procedures and Disorders | 694 | 0.8 | 11,644 | 1.8 | 857 | 0.6 | 8,938 | 1.2 |
| SA Haematological Procedures and Disorders | 887 | 1.0 | 11,959 | 1.8 | 1,611 | 1.1 | 12,997 | 1.7 |
| VA Multiple Trauma | 661 | 0.8 | 3,732 | 0.6 | 2,223 | 1.5 | 7,655 | 1.0 |
| WA_A Immunology, Infectious Diseases, Poisoning | 1,397 | 1.6 | 8,462 | 1.3 | 2,334 | 1.5 | 9,736 | 1.3 |
| WA_B Complications of procedures | 155 | 0.2 | 4,045 | 0.6 | 214 | 0.1 | 3,581 | 0.5 |
| WA_D Unexplained symptoms or abnormal findings | 3,494 | 4.0 | 9,578 | 1.5 | 5,985 | 3.9 | 12,483 | 1.7 |
| RC+WA_C+WA_E+ZZ Miscellaneous | 6,178 | 7.0 | 24,306 | 3.7 | 11,149 | 7.3 | 32,421 | 4.3 |
| missing or N/A “Not applicable” | 3,122 | 3.6 | 21,298 | 3.2 | 5,309 | 3.5 | 23,714 | 3.1 |
| **Post-admission factors** |  |  |  |  |  |  |  |  |
| Number of episodes in spell |  |  |  |  |  |  |  |  |
| 1 | 42,627 | 48.5 | 369,253 | 56.0 | 79,229 | 51.7 | 428,481 | 56.6 |
| 2 | 31,691 | 36.0 | 202,237 | 30.7 | 52,387 | 34.2 | 227,985 | 30.1 |
| 3 | 9,485 | 10.8 | 58,745 | 8.9 | 15,568 | 10.2 | 67,976 | 9.0 |
| 4 or more | 4,121 | 4.7 | 29,380 | 4.5 | 5,947 | 3.9 | 32,041 | 4.2 |
| Discharge destination |  |  |  |  |  |  |  |  |
| Usual residence | 62,870 | 71.5 | 555,422 | 84.2 | 110,366 | 72.1 | 624,197 | 82.5 |
| Other hospital | 5,461 | 6.2 | 36,784 | 5.6 | 8,737 | 5.7 | 46,545 | 6.2 |
| Residential care/care home | 7,822 | 8.9 | 11,903 | 1.8 | 15,955 | 10.4 | 21,795 | 2.9 |
| Died | 10,185 | 11.6 | 48,733 | 7.4 | 14,928 | 9.7 | 52,192 | 6.9 |
| Other/unknown/NA | 1,586 | 1.8 | 6,773 | 1.0 | 3,145 | 2.1 | 11,754 | 1.6 |

| **Table A2. Descriptive statistics for the FY 2012/13 hospital spell sample** | | | | | | | | |
| --- | --- | --- | --- | --- | --- | --- | --- | --- |
|  | **Males** | | | | **Females** | | | |
|  | **PwD** |  | **PwoD** |  | **PwD** |  | **PwoD** |  |
|  | **n** | **%** | **n** | **%** | **n** | **%** | **n** | **%** |
| Total number of spells | 111,846 | 13.7 | 706,090 | 86.3 | 182,956 | 18.9 | 786,434 | 81.1 |
| **Patient demographics** |  |  |  |  |  |  |  |  |
| Age on admission |  |  |  |  |  |  |  |  |
| 65-69 | 5,051 | 4.5 | 142,176 | 20.1 | 3,876 | 2.1 | 118,385 | 15.1 |
| 70-74 | 9,554 | 8.5 | 137,603 | 19.5 | 8,784 | 4.8 | 124,937 | 15.9 |
| 75-79 | 19,295 | 17.3 | 146,204 | 20.7 | 20,763 | 11.3 | 145,725 | 18.5 |
| 80-84 | 29,560 | 26.4 | 134,561 | 19.1 | 41,885 | 22.9 | 158,749 | 20.2 |
| 85-89 | 29,437 | 26.3 | 94,443 | 13.4 | 55,115 | 30.1 | 134,137 | 17.1 |
| 90plus | 18,949 | 16.9 | 51,103 | 7.2 | 52,533 | 28.7 | 104,501 | 13.3 |
| Mean (SD) | 82.7 | 7.0 | 77.4 | 7.9 | 85.4 | 6.8 | 79.5 | 8.4 |
| Ethnicity |  |  |  |  |  |  |  |  |
| White | 101,679 | 90.9 | 635,168 | 90.0 | 168,896 | 92.3 | 712,739 | 90.6 |
| Black African | 260 | 0.2 | 1,715 | 0.2 | 252 | 0.1 | 1,877 | 0.2 |
| Black Caribbean | 1,643 | 1.5 | 6,344 | 0.9 | 1,531 | 0.8 | 6,169 | 0.8 |
| Indian | 1,087 | 1.0 | 10,019 | 1.4 | 1,286 | 0.7 | 9,856 | 1.3 |
| Pakistani | 661 | 0.6 | 6,474 | 0.9 | 595 | 0.3 | 5,932 | 0.8 |
| Bangladeshi | 307 | 0.3 | 2,099 | 0.3 | 152 | 0.1 | 1,429 | 0.2 |
| Black other/mixed | 383 | 0.3 | 1,793 | 0.3 | 377 | 0.2 | 1,887 | 0.2 |
| Asian other/mixed | 525 | 0.5 | 3,754 | 0.5 | 495 | 0.3 | 3,358 | 0.4 |
| Other ethnic group/mixed | 1,155 | 1.0 | 7,537 | 1.1 | 1,701 | 0.9 | 7,744 | 1.0 |
| Unknown | 4,146 | 3.7 | 31,187 | 4.4 | 7,671 | 4.2 | 35,443 | 4.5 |
| Residential IMD quintile (2015) |  |  |  |  |  |  |  |  |
| 1 | 24,907 | 22.3 | 142,185 | 20.1 | 40,307 | 22.0 | 162,949 | 20.7 |
| 2 | 23,858 | 21.3 | 139,251 | 19.7 | 38,637 | 21.1 | 159,407 | 20.3 |
| 3 | 23,090 | 20.6 | 146,324 | 20.7 | 38,181 | 20.9 | 161,867 | 20.6 |
| 4 | 21,238 | 19.0 | 141,519 | 20.0 | 35,205 | 19.2 | 156,435 | 19.9 |
| 5 | 18,148 | 16.2 | 131,307 | 18.6 | 29,761 | 16.3 | 140,257 | 17.8 |
| Missing | 605 | 0.5 | 5,504 | 0.8 | 865 | 0.5 | 5,519 | 0.7 |
| Residential area low income quintile |  |  |  |  |  |  |  |  |
| 1 | 16,771 | 15.0 | 133,420 | 18.9 | 25,449 | 13.9 | 135,431 | 17.2 |
| 2 | 19,239 | 17.2 | 133,774 | 18.9 | 31,378 | 17.2 | 143,039 | 18.2 |
| 3 | 21,344 | 19.1 | 134,248 | 19.0 | 36,112 | 19.7 | 150,805 | 19.2 |
| 4 | 24,400 | 21.8 | 136,132 | 19.3 | 40,879 | 22.3 | 162,140 | 20.6 |
| 5 | 26,758 | 23.9 | 146,351 | 20.7 | 44,114 | 24.1 | 171,378 | 21.8 |
| Missing | 3,334 | 3.0 | 22,165 | 3.1 | 5,024 | 2.7 | 23,641 | 3.0 |
| **Spell characteristics** |  |  |  |  |  |  |  |  |
| Month of admission |  |  |  |  |  |  |  |  |
| March-May | 26,271 | 23.5 | 170,023 | 24.1 | 43,360 | 23.7 | 187,571 | 23.9 |
| June-August | 28,609 | 25.6 | 176,337 | 25.0 | 46,446 | 25.4 | 196,352 | 25.0 |
| September-November | 28,658 | 25.6 | 176,615 | 25.0 | 46,657 | 25.5 | 197,480 | 25.1 |
| December-February | 28,308 | 25.3 | 183,115 | 25.9 | 46,493 | 25.4 | 205,031 | 26.1 |
| Weekend admission (Sat or Sun) |  |  |  |  |  |  |  |  |
| Yes | 30,454 | 27.2 | 168,800 | 23.9 | 50,648 | 27.7 | 192,044 | 24.4 |
| No | 81,392 | 72.8 | 537,290 | 76.1 | 132,308 | 72.3 | 594,390 | 75.6 |
| Source of admission |  |  |  |  |  |  |  |  |
| Usual residence | 104,361 | 93.3 | 675,394 | 95.7 | 169,459 | 92.6 | 750,954 | 95.5 |
| Other hospital | 4,260 | 3.8 | 26,495 | 3.8 | 7,099 | 3.9 | 29,563 | 3.8 |
| Residential care/care home | 2,938 | 2.6 | 2,081 | 0.3 | 5,887 | 3.2 | 3,472 | 0.4 |
| Other/unknown | 287 | 0.3 | 2,120 | 0.3 | 511 | 0.3 | 2,445 | 0.3 |
| **Pre-existing health** |  |  |  |  |  |  |  |  |
| Elixhauser comorbidities |  |  |  |  |  |  |  |  |
| Congestive heart failure | 23,577 | 21.1 | 151,387 | 21.4 | 34,600 | 18.9 | 153,234 | 19.5 |
| Cardiac arrhythmias | 49,246 | 44.0 | 276,138 | 39.1 | 68,270 | 37.3 | 267,139 | 34.0 |
| Valvular disease | 11,532 | 10.3 | 87,440 | 12.4 | 18,390 | 10.1 | 95,757 | 12.2 |
| Pulmonary circulation disorders | 3,657 | 3.3 | 35,836 | 5.1 | 6,434 | 3.5 | 43,775 | 5.6 |
| Peripheral vascular disorders | 12,450 | 11.1 | 93,193 | 13.2 | 11,054 | 6.0 | 56,950 | 7.2 |
| Hypertension | 67,482 | 60.3 | 442,713 | 62.7 | 113,214 | 61.9 | 510,649 | 64.9 |
| Paralysis | 6,552 | 5.9 | 25,274 | 3.6 | 7,057 | 3.9 | 22,862 | 2.9 |
| Other (non-dementia) neurodegenerative disorders | 30,549 | 27.3 | 70,469 | 10.0 | 34,052 | 18.6 | 66,687 | 8.5 |
| Chronic pulmonary disease | 28,474 | 25.5 | 217,852 | 30.9 | 38,770 | 21.2 | 231,414 | 29.4 |
| Diabetes, uncomplicated | 28,731 | 25.7 | 182,799 | 25.9 | 36,930 | 20.2 | 164,121 | 20.9 |
| Diabetes, complicated | 4,569 | 4.1 | 28,818 | 4.1 | 3,694 | 2.0 | 20,072 | 2.6 |
| Hypothyroidism | 7,647 | 6.8 | 38,328 | 5.4 | 29,593 | 16.2 | 118,833 | 15.1 |
| Renal failure | 29,011 | 25.9 | 144,744 | 20.5 | 38,063 | 20.8 | 136,246 | 17.3 |
| Liver disease | 2,779 | 2.5 | 26,636 | 3.8 | 3,088 | 1.7 | 22,074 | 2.8 |
| Peptic ulcer disease excluding bleeding | 2,468 | 2.2 | 18,883 | 2.7 | 2,523 | 1.4 | 15,091 | 1.9 |
| Lymphoma | 974 | 0.9 | 13,829 | 2.0 | 913 | 0.5 | 10,782 | 1.4 |
| Metastatic cancer | 3,036 | 2.7 | 51,849 | 7.3 | 2,623 | 1.4 | 40,196 | 5.1 |
| Solid tumour without metastasis | 14,922 | 13.3 | 129,592 | 18.4 | 8,820 | 4.8 | 75,288 | 9.6 |
| Rheumatoid arthritis/collagen vascular diseases | 3,975 | 3.6 | 31,975 | 4.5 | 11,388 | 6.2 | 68,410 | 8.7 |
| Coagulopathy | 1,939 | 1.7 | 14,299 | 2.0 | 2,093 | 1.1 | 11,816 | 1.5 |
| Obesity | 1,760 | 1.6 | 27,272 | 3.9 | 3,037 | 1.7 | 32,582 | 4.1 |
| Weight loss | 4,873 | 4.4 | 31,023 | 4.4 | 6,384 | 3.5 | 31,925 | 4.1 |
| Fluid and electrolyte disorders | 35,813 | 32.0 | 133,769 | 18.9 | 60,221 | 32.9 | 164,891 | 21.0 |
| Blood loss anemia | 327 | 0.3 | 2,408 | 0.3 | 552 | 0.3 | 2,426 | 0.3 |
| Deficiency anemia | 11,066 | 9.9 | 57,268 | 8.1 | 18,418 | 10.1 | 72,068 | 9.2 |
| Alcohol abuse | 6,580 | 5.9 | 45,404 | 6.4 | 4,078 | 2.2 | 18,135 | 2.3 |
| Drug abuse | 173 | 0.2 | 1,162 | 0.2 | 224 | 0.1 | 960 | 0.1 |
| Psychoses | 2,195 | 2.0 | 5,260 | 0.7 | 4,085 | 2.2 | 6,790 | 0.9 |
| Depression | 13,757 | 12.3 | 43,507 | 6.2 | 28,936 | 15.8 | 74,021 | 9.4 |
| Number of Elixhauser comorbidities |  |  |  |  |  |  |  |  |
| Mean (SD) | 3.7 | 2.3 | 3.4 | 2.3 | 3.3 | 2.2 | 3.2 | 2.2 |
| No. of emergency admissions in previous FY |  |  |  |  |  |  |  |  |
| None | 54,392 | 48.6 | 472,492 | 66.9 | 94,464 | 51.6 | 526,613 | 67.0 |
| 1 | 26,686 | 23.9 | 125,482 | 17.8 | 43,826 | 24.0 | 144,199 | 18.3 |
| 2 | 13,955 | 12.5 | 52,105 | 7.4 | 21,680 | 11.8 | 58,503 | 7.4 |
| 3 | 7,320 | 6.5 | 24,223 | 3.4 | 10,647 | 5.8 | 26,735 | 3.4 |
| 4 | 4,042 | 3.6 | 12,596 | 1.8 | 5,224 | 2.9 | 13,188 | 1.7 |
| 5 | 2,104 | 1.9 | 6,926 | 1.0 | 2,771 | 1.5 | 6,689 | 0.9 |
| 6 | 1,188 | 1.1 | 3,904 | 0.6 | 1,747 | 1.0 | 3,827 | 0.5 |
| 7+ | 2,159 | 1.9 | 8,362 | 1.2 | 2,597 | 1.4 | 6,680 | 0.8 |
| **Admitting condition type and severity** |  |  |  |  |  |  |  |  |
| Spell Core HRG subchapter |  |  |  |  |  |  |  |  |
| AA+AB Nervous System Procedures and Disorders/Pain management | 9,313 | 8.3 | 52,204 | 7.4 | 14,790 | 8.1 | 58,651 | 7.5 |
| BZ Eyes and Periorbita Procedures and Disorders | 364 | 0.3 | 3,202 | 0.5 | 808 | 0.4 | 3,755 | 0.5 |
| CZ Mouth Head Neck and Ears Procedures and Disorders | 3,657 | 3.3 | 20,057 | 2.8 | 7,273 | 4.0 | 22,967 | 2.9 |
| DZ Respiratory System Procedures and Disorders | 22,598 | 20.2 | 129,625 | 18.4 | 29,560 | 16.2 | 133,486 | 17.0 |
| EA Cardiac Procedures | 899 | 0.8 | 22,232 | 3.1 | 726 | 0.4 | 14,433 | 1.8 |
| EB Cardiac Disorders | 12,542 | 11.2 | 120,395 | 17.1 | 18,742 | 10.2 | 126,076 | 16.0 |
| FZ Digestive System Procedures and Disorders | 9,560 | 8.5 | 84,586 | 12.0 | 16,263 | 8.9 | 102,095 | 13.0 |
| GA+GB Hepatobiliary and Pancreatic System Open, Laparoscopic, Endoscopic Procedures | 353 | 0.3 | 6,737 | 1.0 | 538 | 0.3 | 7,008 | 0.9 |
| GC Hepatobiliary and Pancreatic System Disorders | 771 | 0.7 | 13,276 | 1.9 | 1,220 | 0.7 | 13,708 | 1.7 |
| HA Orthopaedic Trauma Procedures | 6,364 | 5.7 | 25,588 | 3.6 | 21,196 | 11.6 | 63,352 | 8.1 |
| HB+HR Orthopaedic Non-Trauma + Reconstruction Procedures | 794 | 0.7 | 7,243 | 1.0 | 1,683 | 0.9 | 9,857 | 1.3 |
| HC Spinal Procedures and Disorders | 730 | 0.7 | 7,442 | 1.1 | 1,638 | 0.9 | 11,507 | 1.5 |
| HD Musculoskeletal and Rheumatological Disorders | 1,823 | 1.6 | 16,540 | 2.3 | 5,161 | 2.8 | 26,724 | 3.4 |
| JA+JB+JC Breast/Burn/Skin Procedures and Disorders | 695 | 0.6 | 5,314 | 0.8 | 1,445 | 0.8 | 6,980 | 0.9 |
| JD Skin Disorders | 1,826 | 1.6 | 14,004 | 2.0 | 3,279 | 1.8 | 17,180 | 2.2 |
| KA+KC Endocrine System/Metabolic Disorders | 1,382 | 1.2 | 6,980 | 1.0 | 3,230 | 1.8 | 10,409 | 1.3 |
| KB Diabetic Medicine | 1,203 | 1.1 | 5,431 | 0.8 | 1,620 | 0.9 | 4,956 | 0.6 |
| LA Renal Procedures and Disorders | 13,425 | 12.0 | 37,932 | 5.4 | 22,446 | 12.3 | 45,229 | 5.8 |
| LB Urological and Male Reproductive System Procedures and Disorders | 6,172 | 5.5 | 41,551 | 5.9 | 1,227 | 0.7 | 7,984 | 1.0 |
| MA+MB Female Reproductive System Procedures/Disorders | NA | NA | NA | NA | 441 | 0.2 | 3,518 | 0.4 |
| QZ Vascular Procedures and Disorders | 1,257 | 1.1 | 16,064 | 2.3 | 2,117 | 1.2 | 13,521 | 1.7 |
| SA Haematological Procedures and Disorders | 1,082 | 1.0 | 12,772 | 1.8 | 1,780 | 1.0 | 13,177 | 1.7 |
| VA Multiple Trauma | 1,117 | 1.0 | 4,759 | 0.7 | 2,919 | 1.6 | 8,771 | 1.1 |
| WA_A Immunology, Infectious Diseases, Poisoning | 2,105 | 1.9 | 11,839 | 1.7 | 3,118 | 1.7 | 12,377 | 1.6 |
| WA_B Complications of procedures | 227 | 0.2 | 4,767 | 0.7 | 250 | 0.1 | 4,077 | 0.5 |
| WA_D Unexplained symptoms or abnormal findings | 4,009 | 3.6 | 9,526 | 1.3 | 6,410 | 3.5 | 11,776 | 1.5 |
| RC+WA_C+WA_E+ZZ Miscellaneous | 5,686 | 5.1 | 14,054 | 2.0 | 10,205 | 5.6 | 19,904 | 2.5 |
| missing or N/A “Not applicable” | 1,874 | 1.7 | 11,633 | 1.6 | 2,844 | 1.6 | 12,638 | 1.6 |
| **Post-admission factors** |  |  |  |  |  |  |  |  |
| Number of episodes in spell |  |  |  |  |  |  |  |  |
| 1 | 51,758 | 46.3 | 384,226 | 54.4 | 90,231 | 49.3 | 432,224 | 55.0 |
| 2 | 41,210 | 36.8 | 220,453 | 31.2 | 64,874 | 35.5 | 243,084 | 30.9 |
| 3 | 13,059 | 11.7 | 67,212 | 9.5 | 19,776 | 10.8 | 75,554 | 9.6 |
| 4 or more | 5,819 | 5.2 | 34,199 | 4.8 | 8,075 | 4.4 | 35,572 | 4.5 |
| Discharge destination |  |  |  |  |  |  |  |  |
| Usual residence | 80,842 | 72.3 | 598,474 | 84.8 | 133,275 | 72.8 | 655,440 | 83.3 |
| Other hospital | 6,254 | 5.6 | 36,537 | 5.2 | 9,541 | 5.2 | 42,230 | 5.4 |
| Residential care/care home | 10,564 | 9.4 | 13,838 | 2.0 | 19,783 | 10.8 | 24,181 | 3.1 |
| Died | 11,958 | 10.7 | 49,257 | 7.0 | 16,200 | 8.9 | 51,445 | 6.5 |
| Other/unknown/NA | 2,228 | 2.0 | 7,984 | 1.1 | 4,157 | 2.3 | 13,138 | 1.7 |

| **Table A3. Summary of group comparisons for all steps of the hierarchical regressions** | | | | | | | |
| --- | --- | --- | --- | --- | --- | --- | --- |
|  | **Males** | | | **Females** | | | |
|  | **2010/11** | **2012/13** | **2016/17** | **2010/11** | **2012/13** | | **2016/17** |
|  | **RR (95% CI)** | **RR (95% CI)** | **RR (95% CI)** | **RR (95% CI)** | **RR (95% CI)** | | **RR (95% CI)** |
| **Length of stay (number of days)** | | | | | | | |
| No control (M1) | 1.52 (1.49-1.55) | 1.50 (1.47-1.53) | 1.55 (1.52-1.58) | 1.37 (1.34-1.40) | 1.36 (1.33-1.39) | | 1.43 (1.40-1.46) |
| Add demographics (M2) | 1.38 (1.35-1.40) | 1.36 (1.33-1.39) | 1.41 (1.39-1.44) | 1.24 (1.21-1.26) | 1.23 (1.21-1.25) | | 1.29 (1.27-1.32) |
| Add spell characteristics (M3) | 1.38 (1.35-1.41) | 1.36 (1.33-1.39) | 1.41 (1.38-1.44) | 1.24 (1.21-1.26) | 1.23 (1.21-1.25) | | 1.29 (1.27-1.32) |
| Add pre-existing health (M4) | 1.35 (1.33-1.38) | 1.33 (1.31-1.36) | 1.38 (1.35-1.41) | 1.24 (1.22-1.27) | 1.24 (1.22-1.26) | | 1.30 (1.27-1.32) |
| Add admitting condition (M5) | 1.31 (1.29-1.33) | 1.29 (1.27-1.32) | 1.35 (1.323-1.38) | 1.20 (1.18-1.22) | 1.19 (1.17-1.21) | | 1.26 (1.23-1.28) |
| Add number of episodes (M6) | 1.23 (1.21-1.26) | 1.21 (1.95-1.23) | 1.24 (1.22-1.27) | 1.15 (1.13-1.17) | 1.14 (1.13-1.16) | | 1.17 (1.16-1.19) |
| Add discharge destination (M7) | 1.15 (1.12-1.17) | 1.13 (1.12-1.15) | 1.17 (1.15-1.18) | 1.09 (1.07-1.11) | 1.09 (1.07-1.10) | | 1.12 (1.10-1.14) |
| **Length of stay>=15 days** | | | | | | | |
| No control (M1) | 1.72 (1.68-1.76) | 1.75 (1.70-1.79) | 1.76 (1.72-1.80) | 1.50 (1.47-1.54) | | 1.54 (1.50-1.57) | 1.59 (1.55-1.63) |
| Add demographics (M2) | 1.45 (1.42-1.49) | 1.47 (1.44-1.51) | 1.50 (1.47-1.53) | 1.27 (1.24-1.29) | | 1.29 (1.27-1.32) | 1.32 (1.30-1.35) |
| Add spell characteristics (M3) | 1.46 (1.43-1.49) | 1.48 (1.44-1.51) | 1.50 (1.47-1.54) | 1.27 (1.24-1.29) | | 1.30 (1.27-1.32) | 1.33 (1.30-1.36) |
| Add pre-existing health (M4) | 1.37 (1.34-1.40) | 1.39 (1.36-1.42) | 1.41 (1.38-1.44) | 1.25 (1.22-1.27) | | 1.27 (1.25-1.30) | 1.30 (1.27-1.32) |
| Add admitting condition (M5) | 1.33 (1.30-1.35) | 1.35 (1.32-1.38) | 1.38 (1.36-1.41) | 1.20 (1.18-1.22) | | 1.22 (1.20-1.25) | 1.26 (1.23-1.28) |
| Add number of episodes (M6) | 1.29 (1.27-1.32) | 1.30 (1.28-1.32) | 1.31 (1.28-1.33) | 1.19 (1.17-1.21) | | 1.21 (1.19-1.23) | 1.21 (1.19-1.24) |
| Add discharge destination (M7) | 1.19 (1.17-1.22) | 1.20 (1.18-1.23) | 1.22 (1.19-1.24) | 1.13 (1.11-1.15) | | 1.15 (1.13-1.17) | 1.16 (1.14-1.18) |
| **Emergency re-admission within 30 days** | | | | | | | |
| No control (M1) | 1.44 (1.40-1.47) | 1.43 (1.41-1.46) | 1.31 (1.28-1.33) | 1.36 (1.34-1.39) | | 1.37 (1.34-1.40) | 1.25 (1.23-1.27) |
| Add demographics (M2) | 1.37 (1.34-1.40) | 1.35 (1.32-1.38) | 1.26 (1.24-1.28) | 1.31 (1.29-1.33) | | 1.31 (1.28-1.33) | 1.22 (1.20-1.24) |
| Add spell characteristics (M3) | 1.37 (1.34-1.40) | 1.35 (1.32-1.38) | 1.26 (1.23-1.28) | 1.31 (1.29-1.33) | | 1.31 (1.29-1.33) | 1.22 (1.20-1.24) |
| Add pre-existing health (M4) | 1.28 (1.25-1.30) | 1.25 (1.23-1.27) | 1.16 (1.15-1.18) | 1.26 (1.24-1.28) | | 1.24 (1.22-1.26) | 1.16 (1.14-1.17) |
| Add admitting condition (M5) | 1.27 (1.25-1.30) | 1.24 (1.22-1.26) | 1.16 (1.14-1.17) | 1.26 (1.24-1.28) | | 1.24 (1.22-1.26) | 1.16 (1.14-1.17) |
| Add number of episodes (M6) | 1.27 (1.25-1.30) | 1.24 (1.22-1.26) | 1.16 (1.14-1.17) | 1.26 (1.24-1.28) | | 1.24 (1.22-1.26) | 1.16 (1.14-1.17) |
| Add discharge destination (M7) | 1.29 (1.26-1.31) | 1.25 (1.23-1.27) | 1.17 (1.16-1.19) | 1.28 (1.26-1.30) | | 1.25 (1.23-1.27) | 1.17 (1.16-1.19) |
| **Death (in hospital or within 30 days of discharge)** | | | | | | | |
| No control (M1) | 1.69 (1.65-1.72) | 1.68 (1.65-1.72) | 1.73 (1.69-1.76) | 1.63 (1.60-1.66) | | 1.60 (1.56-1.63) | 1.74 (1.71-1.77) |
| Add demographics (M2) | 1.33 (1.31-1.36) | 1.33 (1.30-1.35) | 1.36 (1.34-1.39) | 1.28 (1.25-1.30) | | 1.25 (1.22-1.27) | 1.34 (1.32-1.36) |
| Add spell characteristics (M3) | 1.32 (1.29-1.35) | 1.31 (1.28-1.33) | 1.34 (1.32-1.37) | 1.26 (1.24-1.29) | | 1.23 (1.20-1.25) | 1.32 (1.30-1.35) |
| Add pre-existing health (M4) | 1.38 (1.35-1.41) | 1.37 (1.34-1.40) | 1.37 (1.34-1.39) | 1.33 (1.31-1.36) | | 1.30 (1.27-1.33) | 1.39 (1.36-1.41) |
| Add admitting condition (M5) | 1.34 (1.32-1.37) | 1.34 (1.31-1.37) | 1.33 (1.31-1.36) | 1.33 (1.30-1.36) | | 1.30 (1.27-1.32) | 1.37 (1.34-1.39) |
| Add number of episodes (M6) | 1.33 (1.31-1.36) | 1.32 (1.30-1.35) | 1.30 (1.28-1.33) | 1.32 (1.30-1.35) | | 1.29 (1.27-1.32) | 1.35 (1.33-1.38) |
| **Death in hospital** | | | | | | | |
| No control (M1) | 1.57 (1.53-1.61) | 1.53 (1.50-1.57) | 1.53 (1.50-1.57) | 1.41 (1.38-1.45) | | 1.35 (1.32-1.39) | 1.43 (1.40-1.46) |
| Add demographics (M2) | 1.23 (1.20-1.26) | 1.19 (1.16-1.22) | 1.20 (1.17-1.22) | 1.11 (1.09-1.14) | | 1.05 (1.03-1.08) | 1.10 (1.08-1.12) |
| Add spell characteristics (M3) | 1.22 (1.19-1.25) | 1.17 (1.15-1.20) | 1.18 (1.16-1.21) | 1.10 (1.07-1.13) | | 1.04 (1.01-1.06) | 1.09 (1.06-1.11) |
| Add pre-existing health (M4) | 1.25 (1.22-1.28) | 1.21 (1.18-1.24) | 1.19 (1.16-1.22) | 1.16 (1.13-1.19) | | 1.09 (1.06-1.12) | 1.14 (1.11-1.16) |
| Add admitting condition (M5) | 1.23 (1.20-1.26) | 1.19 (1.16-1.22) | 1.15 (1.13-1.18) | 1.16 (1.13-1.19) | | 1.09 (1.07-1.12) | 1.12 (1.10-1.15) |
| Add number of episodes (M6) | 1.22 (1.19-1.25) | 1.18 (1.15-1.21) | 1.14 (1.11-1.16) | 1.16 (1.13-1.19) | | 1.09 (1.07-1.12) | 1.11 (1.09-1.14) |
| **Death within 30 days after discharge** | | | | | | | |
| No control (M1) | 2.26 (2.17-2.35) | 2.33 (2.23-2.43) | 2.48 (2.38-2.58) | 2.64 (2.53-2.75) | | 2.53 (2.42-2.64) | 2.86 (2.77-2.96) |
| Add demographics (M2) | 1.85 (1.78-1.93) | 1.87 (1.80-1.95) | 1.97 (1.90-2.05) | 2.11 (2.03-2.20) | | 1.98 (1.89-2.06) | 2.19 (2.12-2.26) |
| Add spell characteristics (M3) | 1.83 (1.75-1.91) | 1.84 (1.76-1.91) | 1.92 (1.84-2.00) | 2.08 (2.00-2.17) | | 1.93 (1.85-2.02) | 2.13 (2.06-2.21) |
| Add pre-existing health (M4) | 2.02 (1.94-2.12) | 2.02 (1.94-2.11) | 2.05 (1.96-2.13) | 2.23 (2.14-2.33) | | 2.09 (2.00-2.19) | 2.30 (2.22-2.38) |
| Add admitting condition (M5) | 1.97 (1.88-2.06) | 1.97 (1.89-2.05) | 1.99 (1.91-2.08) | 2.20 (2.12-2.30) | | 2.08 (1.98-2.17) | 2.27 (2.19-2.35) |
| Add number of episodes (M6) | 1.94 (1.85-2.03) | 1.93 (1.85-2.01) | 1.94 (1.86-2.02) | 2.20 (2.11-2.29) | | 2.06 (1.97-2.16) | 2.23 (2.15-2.31) |
| **Emergency re-admission or death** | | | | | | | |
| No control (M1) | 1.48 (1.46-1.50) | 1.48 (1.46-1.50) | 1.39 (1.38-1.41) | 1.42 (1.40-1.43) | | 1.42 (1.40-1.44) | 1.36 (1.35-1.38) |
| Add demographics (M2) | 1.32 (1.31-1.34) | 1.31 (1.30-1.33) | 1.27 (1.26-1.28) | 1.27 (1.26-1.28) | | 1.27 (1.25-1.28) | 1.25 (1.23-1.26) |
| Add spell characteristics (M3) | 1.31 (1.30-1.33) | 1.31 (1.29-1.32) | 1.26 (1.25-1.28) | 1.26 (1.25-1.28) | | 1.26 (1.25-1.28) | 1.24 (1.23-1.25) |
| Add pre-existing health (M4) | 1.29 (1.27-1.31) | 1.27 (1.26-1.29) | 1.22 (1.21-1.23) | 1.27 (1.25-1.28) | | 1.25 (1.24-1.27) | 1.22 (1.21-1.23) |
| Add admitting condition (M5) | 1.28 (1.26-1.30) | 1.26 (1.25-1.28) | 1.21 (1.20-1.22) | 1.27 (1.25-1.28) | | 1.25 (1.24-1.27) | 1.22 (1.21-1.23) |
| Add number of episodes (M6) | 1.28 (1.26-1.29) | 1.26 (1.24-1.27) | 1.20 (1.19-1.22) | 1.27 (1.25-1.28) | | 1.25 (1.24-1.26) | 1.22 (1.21-1.23) |
| RR = Relative Risk | | | | | | | |

| **Table A4. Summary of sensitivity analyses for hospital outcomes 2016/17** | | | | | | |
| --- | --- | --- | --- | --- | --- | --- |
|  | **Excluding LoS<2** | **Excluding spells with psychiatric comorbidities** | **Matched samples** | **Two-level random effects model** | **Logistic model^b^** | **Competing risks model** |
|  | **RR (95% CI)** | **RR (95% CI)** | **RR (95% CI)** | **RR (95% CI)** | **RR (95% CI)** | **SHR^c^ (95% CI)** |
| **Males** | | | | | | |
| **Length of stay (count)** |  |  |  |  |  |  |
| Adjusted for pre-admission factors (M5) | 1.41 (1.37-1.44) | 1.42 (1.39-1.45) | 1.32 (1.30-1.34) | 1.14 (1.14-1.15) | - | 1.14 (1.12-1.15) |
| Adjusted for pre- and post-admission factors (M7) | 1.23 (1.19-1.24) | 1.20 (1.18-1.22) | 1.15 (1.14-1.17) | 1.09 (1.08-1.09) | - | 1.11 (1.10-1.12) |
| **Length of stay>=15 days** | | | | | | |
| Adjusted for pre-admission factors (M5) | 1.32 (1.30-1.34) | 1.47 (1.44-1.50) | 1.40 (1.36-1.44) | 1.38 (0.89-2.15) | 1.38 (1.36-1.41) | - |
| Adjusted for pre- and post-admission factors (M7) | 1.20 (1.18-1.22) | 1.27 (1.24-1.29) | 1.24 (1.21-1.27) | 1.22 (1.05-1.41) | 1.21 (1.19-1.23) | - |
| **Emergency re-admission within 30 days** | | | | | | |
| Adjusted for pre-admission factors (M5) | 1.15 (1.14-1.17) | 1.19 (1.17-1.21) | 1.24 (1.21-1.26) | 1.16 (1.13-1.19) | 1.16 (1.14-1.17) | - |
| Adjusted for pre- and post-admission factors (M7) | 1.17 (1.15-1.18) | 1.20 (1.18-1.22) | 1.25 (1.22-1.28) | 1.17 (1.15-1.18) | 1.17 (1.16-1.18) | - |
| **Death** | | | | | | |
| Adjusted for pre-admission factors (M5) | 1.33 (1.31-1.36) | 1.38 (1.35-1.41) | 1.32 (1.28-1.36) | 1.33 (1.29-1.37) | 1.33 (1.30-1.35) | - |
| Adjusted for pre- and post-admission factors (M6) | 1.28 (1.26-1.31) | 1.35 (1.32-1.38) | 1.30 (1.26-1.33) | 1.30 (1.18-1.44) | 1.30 (1.28-1.33) | - |
| **Emergency re-admission or death** | | | | | | |
| Adjusted for pre-admission factors (M5) | 1.21 (1.20-1.22) | 1.25 (1.23-1.26) | 1.26 (1.24-1.27) | 1.21 (1.03-1.42) | 1.21 (1.20-1.22) | - |
| Adjusted for pre- and post-admission factors (M6) | 1.20 (1.18-1.21) | 1.24 (1.23-1.26) | 1.25 (1.23-1.27) | 1.20 (1.06-1.37) | 1.20 (1.19-1.21) | - |
| **Females** | | | | | | |
| **Length of stay (count)** | | | | | | |
| Adjusted for pre-admission factors (M5) | 1.28 (1.26-1.31) | 1.32 (1.29-1.35) | 1.22 (1.21-1.23) | 1.09 (1.09-1.10) | - | 1.11 (1.10-1.12) |
| Adjusted for pre- and post-admission factors (M7) | 1.17 (1.15-1.19) | 1.15 (1.13-1.17) | 1.11 (1.10-1.12) | 1.06 (1.05-1.06) | - | 1.08 (1.07-1.09) |
| **Length of stay>=15 days** | | | | | | |
| Adjusted for pre-admission factors (M5) | 1.23 (1.20-1.25) | 1.32 (1.29-1.35) | 1.25 (1.23-1.28) | 1.26 (0.91-1.73) | 1.26 (1.23-1.28) | - |
| Adjusted for pre- and post-admission factors (M7) | 1.15 (1.14-1.17) | 1.20 (1.17-1.22) | 1.17 (1.14-1.19) | 1.16 (0.95-1.42) | 1.15 (1.13-1.17) | - |
| **Emergency re-admission within 30 days** | | | | | | |
| Adjusted for pre-admission factors (M5) | 1.15 (1.13-1.16) | 1.19 (1.17-1.21) | 1.20 (1.17-1.23) | 1.16 (0.81-1.67) | 1.16 (1.14-1.17) | - |
| Adjusted for pre- and post-admission factors (M7) | 1.17 (1.15-1.18) | 1.21 (1.19-1.23) | 1.21 (1.19-1.24) | 1.17 (0.87-1.58) | 1.17 (1.16-1.19) | - |
| **Death** | | | | | | |
| Adjusted for pre-admission factors (M5) | 1.35 (1.33-1.38) | 1.41 (1.38-1.43) | 1.35 (1.32-1.38) | 1.37 (1.32-1.42) | 1.37 (1.34-1.39) | - |
| Adjusted for pre- and post-admission factors (M6) | 1.33 (1.30-1.35) | 1.39 (1.36-1.42) | 1.34 (1.30-1.38) | 1.36 (1.16-1.59) | 1.35 (1.33-1.38) | - |
| **Emergency re-admission or death** | | | | | | |
| Adjusted for pre-admission factors (M5) | 1.22 (1.20-1.23) | 1.26 (1.25-1.28) | 1.24 (1.23-1.26) | 1.22 (0.82-1.82) | 1.22 (1.21-1.23) | - |
| Adjusted for pre- and post-admission factors (M6) | 1.21 (1.20-1.22) | 1.26 (1.25-1.27) | 1.24 (1.23-1.25) | 1.22 (0.84-1.77) | 1.22 (1.21-1.23) | - |
| NA = Not applicable; RR= Relative Risk  ^b^For the logistic models, the relative risk is estimated from the odds-ratio returned by the model  ^c^SHR = sub-hazard ratio | | | | | | |

**
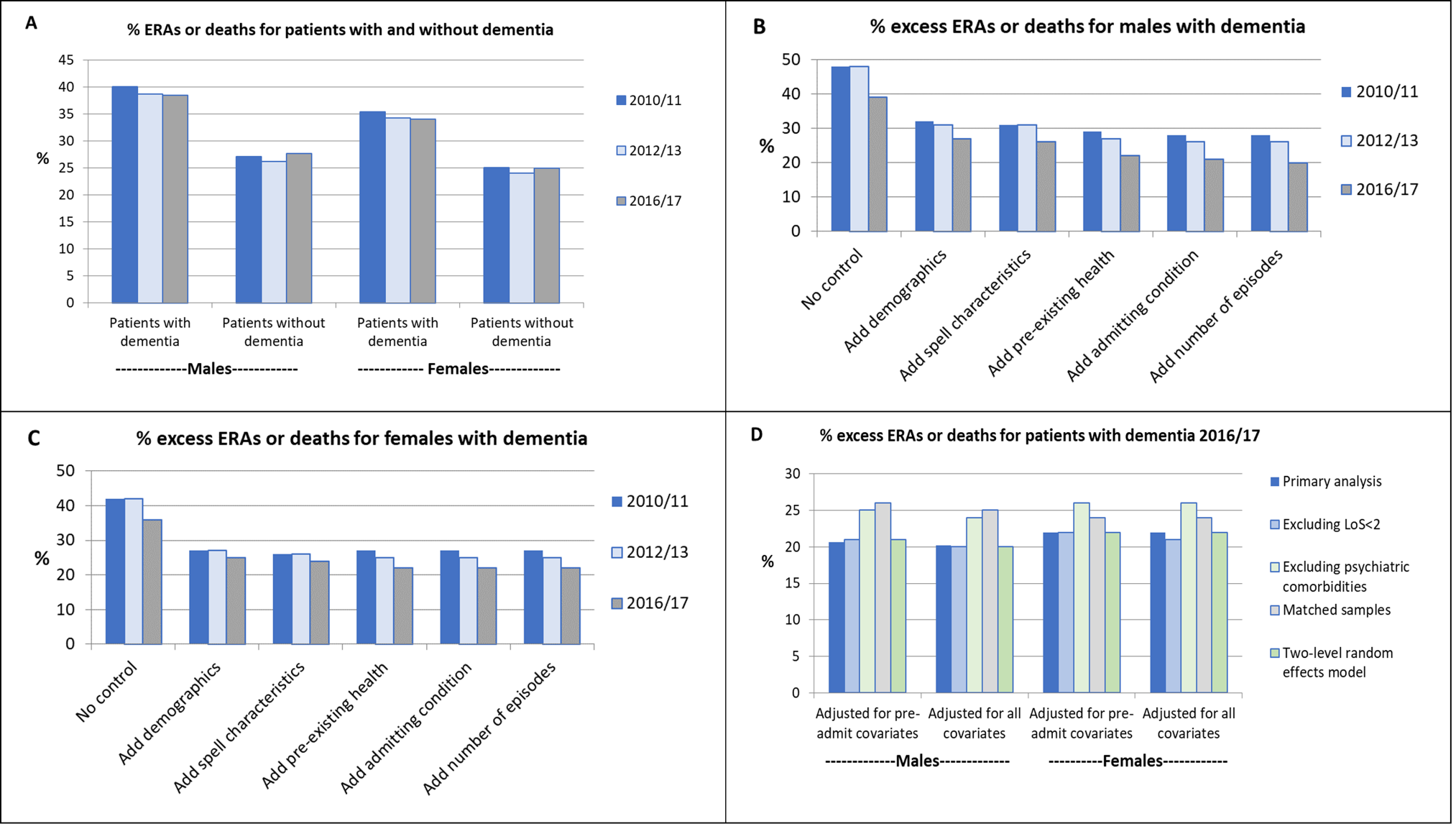
**

**Fig A1. Summary of analysis results for ERA or death as a combined outcome**

**Fig A2. Comparison of results from the primary and sensitivity analyses FY 2016/17**

**Details of sensitivity analyses**

Exclusion of stays of <2 days. Some previous studies have excluded admissions of less than one or two days,[1-3], assuming for example these might be mis-coded day cases; we therefore conducted sensitivity analysis excluding spells of 0 or 1 day. For LoS as an outcome, these analyses used a left-truncated negative binomial model.

Exclusion of spells with psychiatric comorbidities. We observed substantial associations between a diagnosis of dementia and comorbidities of other neurodegenerative disorders, depression and psychosis. Such relationships are well-known,[4] but these conditions can be confused with dementia[5] and might constitute mis- or double-coding. Sensitivity analysis was conducted excluding spells with these comorbidities from both patient groups, to provide a comparison between patients with physical comorbidities only.

Analysis of a matched sample. We selected a matched sample of spells for PwD and PwoD at a 1:1 ratio. Matching was done within FY and hospital. Each spell for a PwD was paired with a random spell for a PwoD of the same: sex, age-band, number of EAs in the prior year (0-7+), number of Elixhauser conditions (within +/- 2) and admitted for the same primary procedure (based on HRG chapter). Matching was with replacement and around 10% of spells for PwD that could not be matched were dropped. For 2016/17 the matched sample consisted of 137,105 spells for male PwD and 203,080 for female PwD, plus the same numbers for PwoD.

For binary outcomes a Conditional Poisson regression model was utilised;[6] for LoS an Unconditional Negative Binomial model was applied.[7] Both matched and unmatched covariates were included in the regression models.[8]

Use of alternative statistical models. We re-analysed each outcome using a two-level random-effects model (Stata commands xtnbreg or xtpoisson) to accommodate between-hospital differences. These models often failed to converge due to the model complexity and large sample sizes, but we were able to achieve convergence for most models using a 50% random subsample of all spells. We report results where convergence was achieved.

We re-analysed length of stay using a Fine and Gray competing risks survival model, to account for death in hospital as a competing risk.[9] We also re-analysed binary outcomes using logistic regression in place of Poisson and converted the resulting odds-ratios to relative risks to facilitate comparisons to the Poisson results.[10]

**References**

1. CHKS. Insight report. An economic analysis of the excess costs for acute care for patients with dementia. Basingstoke: 2013.

2. Hapca S, Guthrie B, Cvoro V, Bu F, Rutherford AC, Reynish E, et al. Mortality in people with dementia, delirium, and unspecified cognitive impairment in the general hospital: prospective cohort study of 6,724 patients with 2 years follow-up. Clinical epidemiology. 2018;10:1743-53. Epub 2018/12/13. doi: 10.2147/clep.s174807. PubMed PMID: 30538578; PubMed Central PMCID: PMCPMC6257080.

3. Sampson EL, Leurent B, Blanchard MR, Jones L, King M. Survival of people with dementia after unplanned acute hospital admission: a prospective cohort study. Int J Geriatr Psychiatry. 2013;28(10):1015-22. Epub 2013/01/03. doi: 10.1002/gps.3919. PubMed PMID: 23280594.

4. Onyike CU. Psychiatric Aspects of Dementia. Continuum (Minneap Minn). 2016;22(2 Dementia):600-14. doi: 10.1212/CON.0000000000000302. PubMed PMID: 27042910.

5. Cerejeira J, Lagarto L, Mukaetova-Ladinska EB. Behavioral and psychological symptoms of dementia. Frontiers in neurology. 2012;3:73. Epub 2012/05/16. doi: 10.3389/fneur.2012.00073. PubMed PMID: 22586419; PubMed Central PMCID: PMCPMC3345875.

6. Cummings P, McKnight B. Analysis of matched cohort data. Stata Journal. 2004;4(3):274-81.

7. Allison PD, Waterman R. Fixed effects negative binomial regression models. In: Stolzenberg RM, editor. Sociological Methodology. Oxford: Basil Blackwell; 2002. p. 247-65.

8. Sjölander A, Greenland S. Ignoring the matching variables in cohort studies - when is it valid and why? Statistics in medicine. 2013;32(27):4696-708. Epub 2013/06/14. doi: 10.1002/sim.5879. PubMed PMID: 23761197.

9. Fogg C, Meredith P, Culliford D, Bridges J, Spice C, Griffiths P. Cognitive impairment is independently associated with mortality, extended hospital stays and early readmission of older people with emergency hospital admissions: A retrospective cohort study. International Journal of Nursing Studies. 2019;96:1-8. doi: <https://doi.org/10.1016/j.ijnurstu.2019.02.005>.

10. Austin PC. Absolute risk reductions, relative risks, relative risk reductions, and numbers needed to treat can be obtained from a logistic regression model. Journal of Clinical Epidemiology. 2010;63(1):2-6. doi: <https://doi.org/10.1016/j.jclinepi.2008.11.004>.
